# Exposure risk management: Personal protective equipment and the risk of accidents occurring during aerosol generating procedures applied to COVID-19 patients

**DOI:** 10.1371/journal.pone.0282673

**Published:** 2023-03-07

**Authors:** Ștefan Andrei Neştian, Silviu-Mihail Tiţă, Elena-Sabina Turnea, Oana Stanciu, Vladimir Poroch

**Affiliations:** 1 Department of Management, Marketing and Business Administration, Alexandru Ioan Cuza University of Iași, Iași, Romania; 2 Faculty of Medicine and Biological Sciences, University “Stefan cel Mare”, Suceava, Romania; 3 Grigore T. Popa University of Medicine and Pharmacy, Iași, Romania; Institute of Advanced Materials, IAAM, SWEDEN

## Abstract

**Background:**

COVID-19 is considered to be very contagious as it can be spread through multiple ways. Therefore, exposure risk of healthcare workers (HCWs) treating COVID-19 patients is a highly salient topic in exposure risk management. From a managerial perspective, wearing personal protective equipment and the risk of accidents occurring during aerosol generating procedures applied to COVID-19 patients are two interconnected issues encountered in all COVID-19 hospitals.

**Objective:**

The study was conducted to understand the realistic impact of exposure risk management on HCWs exposed to risks of SARS-CoV-2 virus infection in a healthcare unit. In particular, this study discusses the role of personal protective equipment (PPEs) used in aerosol generating procedures (AGPs) to protect HCWs, and the related risk of accidents occurring when performing AGPs.

**Methodology:**

This is a cross-sectional single-hospital study conducted at the “Sf. Ioan cel Nou” Hospital in Suceava, Romania, that had to ensure safety of healthcare workers (HCWs) getting in contact with COVID-19 cases. Data used in the study were collected between 10.12.2020–19.03.2021 by means of a questionnaire that collected information on risk assessment and healthcare workers’ exposure management, and which was translated and adapted from the World Health Organization (WHO) and applied to respondents online. For this purpose, ethical approval was obtained, doctors and nurses from all hospital departments being invited to complete the questionnaire. Data processing, as well as descriptive, correlation and regression analyses have been done by using the 21.0 version of the Statistical Package for Social Sciences software.

**Results:**

Most of the 312 HCWs reported having always used disposable gloves (98.13%), medical masks N95 (or equivalent) (92.86%), visors or googles (91.19%), disposable coverall (91.25%) and footwear protection (95.00%) during AGPs. The waterproof apron had always been worn only by 40% of the respondents, and almost 30% of staff had not used it at all during AGPs. Over the last three months, the period when the questionnaire was completed, 28 accidents were reported while performing AGPs: 11 accidents with splashing of biological fluids/ respiratory secretions in the eyes, 11 with splashing of biological fluids/ respiratory secretions on the non-idemn skin, 3 with splashing of biological fluids/ respiratory secretions in the oral/ nasal mucosa and 3 with puncture/ sting with any material contaminated with biological fluids/ respiratory secretions. Also, 84.29% of respondents declared having changed their routine, at least, moderately due to COVID-19.

**Conclusion:**

An effective risk exposure management is based on wearing protective equipment. The only protection offered by the disposable coverall, as it results from our analysis, is related to splashing of biological fluids/ respiratory secretions on the non-idemn skin. In addition, the results show that the number of accidents should decrease due to the fact that disposable gloves and footwear protection are used while performing AGPs on patients with COVID-19 and hand hygiene is practised before and after touching a patient with COVID-19 (regardless of glove wearing).

## 1. Introduction

Because of the high number of COVID-19 infections reported across the globe, on March 11, 2020, was declared the pandemic [1]. For this reason, many public health centres and hospitals were opened for treating COVID-19 [2].

COVID-19 can be spread through aerosol/aerated solids, fluids from human secretions, droplets from normal breathing, coughing, sneezing and surface contact, and therefore is considered very contagious [3]. According to Wong SCY et al. (2020), SARS-CoV-2 is not spread by an airborne route [4]. So, one of the largest Chinese medicine hospitals in China, the Guangdong Provincial Hospital of Chinese Medicine, set in January 2020 a set of prevention rules aimed at avoiding hospital-related infections during the COVID-19 epidemic [5] in order to ensure the protection of healthcare workers (HCWs) during Aerosol generating procedures (AGPs). For this purpose, HCWs wore the following personal protective equipment (PPE): N95 respirators, goggles or protective screens, latex gloves, and medical protective clothing [5] for such procedures as bronchoscopy, tracheal intubation, sputum suction and aerosol or splash operations.

Although a lot of effort was put into vaccine introduction and use, COVID-19 still has an impact on healthcare services, while personnel working in medical systems are at higher risk of infection [6, 7]. Frontline HCWs remain at high risk of getting the COVID-19 infection [8], despite all the effort made to limit virus spreading, therefore it has become essential to perform real-time activities monitoring, apply immediate corrections and pay more constant attention to infection prevention than the treatment itself [6, 9, 10]. As it has been reported, 90000 HCWs across the globe had been infected with COVID-19 in the first year of the pandemic [8]. So, it is crucial for HCWs to wear an appropriate PPE and actively participate in prevention programmes [11] as it has been widely recognised that infected patients are the main cause for COVID-19 transmission [12].

This study was conducted using a questionnaire translated and adapted from the World Health Organization (WHO) [13] with the purpose of understanding the realistic impact of exposure risk management on HCWs exposed to risks of SARS-CoV-2 virus infection in a healthcare unit. In particular, this paper discusses the role of personal protective equipment (PPEs) used in aerosol generating procedures (AGPs) to protect HCWs, and the related risk of accidents occurring when performing AGPs. In this regard, 312 confidential answers were collected online between 10.12.2020–19.03.2021 from medical doctors and nurses at the “Sf. Ioan cel Nou” County Emergency Hospital in Suceava, Romania. The ethical approval has been obtained for this study.

## 2. Literature review

### 2.1. AGPs applied to COVID-19 patients

Aerosols are small liquids or solid particles present in the air, ranging from 0.001 μm to 100 μm [14]. Small particles can live at far distance for a long period of time, these are named “airborne”, the bigger aerosols being named “droplets” [14].

Nurses have been exposed to COVID-19 since they have a direct contact with COVID-19 patients, especially in the context of AGPs [9, 12]. In China, HCWs infection with this virus was associated with aerosol transmission and high-concentration pathogen aerosol environments [12]. Lately, such procedures as suctioning before intubation, and auxiliary intubation have been classified as high-risk activities [12]. Another source explains that some AGPs made on unprotected COVID-19 patients have led to SARS-CoV-2 HCWs’ infection [15]. There is a concern that this virus can be transmitted even during talking and breathing while AGPs are performed [16]. While the Chinese National Hygiene and Health Committee confirmed the possibility of getting the COVID-19 infection through aerosols [14], according to Mahmood et al. (2020), when AGPs are performed, the virus spreads irrespective whether patients present or not COVID-19 symptoms [17].

In Zhongnan Hospital of Wuhan University, out of 103 infected employees, before getting the infection, almost half worked over 7 hours per day in a COVID-19 contaminated environment [18]. In the same hospital, aerosol transmission was cited among top three ways of getting the infection [18].

An adequate PPE is strongly recommended against the risk of HCW getting infected with COVID-19 [9, 19]. To avoid any contamination with COVID-19 during aerosols, PPE is recommended for eyes, face and head protection, together with reusable equipment (glasses, visors or face shields) [17, 20]. A study of 85% of HCWs being involved in AGPs showed that hand hygiene and PPE were the measures having protected them against COVID-19 infection [9]. WHO recommends that HCWs use PPE considering the AGPs types [12]. HCWs should wear a gown, gloves, respirator N95, an apron, and eye protection when they apply AGPs to COVID-19 patients [12]. In the absence of AGPs, the apron and the respirator are not necessary, and the respirator should be replaced by a medical mask [12]. According to the Emergency Medical Services Commission, to perform intubation or cardiopulmonary resuscitation, which are considered AGPs, PPE should mandatorily contain a N95 mask [21].

One study conducted in Australia, identified such barriers as supplier availability, workplace rationing, delivery delays, high costs, no PPE supply at the workplace, denial of PPE based on a low perception of risk or inappropriateness by management [22]. The major risk in AGP is nosocomial infections [23].

### 2.2. PPE during AGPs applied to COVID-19 patients

PPE has fulfilled for over 100 years its main purpose of protecting the patient and the surgeon, and it includes: eye protection, gowns, gloves, surgical face masks, non-powered filtering face piece respirators, and powered air-purifying respirators [24]. Full PPE for HCWs should contain face shields, goggles, masks, coverall/ gowns, head covers, and shoe covers [25]. According to the Minister of Health ORDER no. 533 of March 29, 2020 in Romania, medical staff involved in direct patient care must use the following PPE: gowns, gloves, mask and eye protection (goggles or face shield) [26]. Specifically, in case of AGPs performed for patients with COVID-19 (for example, intubation, non-invasive ventilation, tracheostomy, cardiopulmonary resuscitation, manual ventilation before intubation, bronchoscopy, gastroscopy and collection of PCR COVID tests), medical staff must use the following protection: gloves, gowns, FFP2 and FFP3 masks, and waterproof aprons if the coveralls/gowns are not waterproof [26].

In the context of COVID-19, wearing different pieces of PPE has become very questionable [24]. However, it has been proven that this virus can be transmitted especially through contact with infected patients without wearing PPE [12]. Wearing a PPE is not always the best way of getting protected against COVID-19. For example, it has been stated that eye googles are less beneficial in reducing exposure risks when treating COVID-19 patients than face masks and coverall [3]. In a study conducted by Al Youha et al. (2021), it was found that reuse of PPE is positively correlated with risk infections [6]. In a study made on HCWs at the Zhongnan Hospital of Wuhan University, out of 105 HCWs infected with COVID-19, 43 saw PPE problems as being the cause of their infection [18]. Compared to the times before the outbreak, a focus definitely was on wearing special PPE comprising: clothing, googles, a face shield and also isolation clothing [18]. In another study conducted on 120 HCWs at the First Affiliated Hospital of Chongqing Medical University in China, the following PPE weaknesses during pandemic were reported: inappropriate sizes, uncomfortable design, complexity of use, doubts related to the quality and effectiveness of PPE [27].

During this pandemic, PPE supply was a concern. In a busy tertiary care hospital in the United Kingdom, a negative factor in the fight against the virus was the supply of PPE for HCWs [28]. In a study conducted at the Patan Hospital in Nepal, out of t 58 respondents, 32.7% reported having felt more comfortable in non-Covid emergencies [29]. Also, in the same study, HCWs from non-Covid emergencies reported having felt safer in the presence of PPE wearing [29]. Other case-study in India observed that a breach in masking had been associated with a positivity of 9.6% in all exposures and 33.3% in the high-risk exposure group among HCWs, while a breach in goggles wearing had been associated with a total and high-risk exposure COVID-19 positivity of 4.6% and 22.2%, respectively [30].

The case of South America was analysed in one study. The rates of SARS-CoV-2 infection among HCW in São Paulo city ranged from 5.5% (IgG ELISA) in a private hospital to 14% (IgG/IgM antibody, WONDFO) in a large public hospital in 2020 due to adoption of high-quality hospital infection control and provision of complete PPE in the early stages of the COVID-19 pandemic [31].

Not only PPE is important during AGPs, it seems that HCWs’ hand hygiene is also significant, and should be monitored [32–34]. In this regard, the five times during the day when hand hygiene should be complied with are: before touching a patient, cleaning/ aseptic procedures, after performing procedures/ body fluid exposures, after touching a patient, or a patient’s surroundings [34].

### 2.3. Accidents occurring during AGPs applied to COVID-19 patients and ways of protecting HCWs

In a study conducted on 80 frontline nurses in COVID-19 hospitals in the north of Saudi Arabia, 3.8% of the respondents reported such accidents related to biological material handling as splashes of a biological fluid (into the eyes) [9]. In a study conducted at the Jaber Al Ahmad Hospitali n Kuwait comprising 847 HCWs, it was found that working as a nurse and wearing gloves had been strongly associated with an increased likelihood of contracting SARS-CoV-2 infection, while monitoring other factors [6]. Also, the same study reported that PPE had been available most of the time in the hospital, coveralls being the least available [6]. Other study showed that risk for the first responder workforce primarily originated from non-patient sources; 29 of 30 COVID-19 illnesses among Emergency Medical Services providers had not been directly attributed to COVID-19 patient encounters, and collectively, the involvement of such a large proportion of the first responder conducted in the King County, Washington, workforce, the heterogeneous nature of patient characteristics, and the time-pressured need among some patients for AGP intervention could pose major COVID-19 risk to public safety personnel and infrastructure [35].

In laboratories, SARS-CoV-2 aerosols live on average 5.6 hours on stainless steel, and 6.8 hours on plastic [24]. The number of healthcare-associated infections can be reduced among HCWs by adopting a proper hygiene compliance, constant audits and timely feedback to prevent infections [7, 34]. The United States Centres for Disease Control and Prevention strongly recommend the use of eye protection when contacting a patient suspected of having COVID-19 [24]. The surgical N95 masks are also helpful in triages and during AGPs as they prevent getting the infection [24]. But the reuse of N95 masks is questionable as there has been so far limited research on this issue [25]. If medical surgical masks are correctly worn, they can block particles exceeding 5μm, which can curb the spread of droplets [10]. These should be replaced once contaminated [10]. HCWs have to ensure that there is no gap between the mask and the face [3].

Face shields are recommended as a substitute to goggles, and offer protection against splashes and splatter of aerosols on a larger area of face [25]. Goggles are extremely important in protecting the exposed conjunctiva against infected droplets and aerosols from patients, while aprons, gowns and coveralls offer full protection to HCWs [10, 25]. In high-risk areas, it is recommended that two or even three layers of surgical gloves be worn [10]. Poor glove usage by nurses can bring COVID-19 infections of HCWs [6]. Also, HCWs should pay attention to prolonged glove use as it is associated with higher likelihood of SARS-CoV-2 infection [6].

In procedures involving splashing of body fluids, blood, etc., use of goggles and protective face shields/ face screens are mandatory [10]. Shoe covers should be worn when entering into a contaminated area [10]. The coverall gown is highly efficient in protecting HCWs from airborne pathogens, including aerosols/ aerated solids [3]. Despite the protection that N95 masks and face shields offer, the coveralls play a key role in protecting HCWs from the virus [3]. When AGPs are performed, air isolation measures, frequent ventilation and powered air-purifying respirators are also needed to prevent against COVID-19 infection [12].

To formulate the research hypotheses, this study started from data provided by earlier studies, which have been described above, following three main research lines: AGPs applied to COVID-19 patients, PPE during AGPs applied to COVID-19 patients, and accidents occurring during AGPs for COVID-19 patients and ways of protecting HCWs. In the first phase, the assumption was made that when performing AGPs, the coverall is worn with all the other accessories for ensuring the highest protection. Therefore, the following H1 hypothesis was formulated:

*H1*. *There is a positive correlation between wearing disposable coverall when performing aerosol generating procedures (AGPs) on a COVID-19 patient and wearing disposable gloves*, *medical masks N95 (or equivalent)*, *visor or goggles*, *and footwear protection*.

Also, it has been assumed that the waterproof apron is worn during AGPs with all the other accessories for providing the highest protection. So, the following H2 hypothesis was formulated:

*H2*. *There is a positive correlation between wearing waterproof apron when performing aerosol generating procedures (AGPs) on a COVID-19 patient and wearing disposable gloves*, *medical masks N95 (or equivalent)*, *visor or goggles*, *disposable coverall*, *and footwear protection*.

We started from the premise that coverall equipment, which offers the highest and complete protection, should lead to a decrease in accident frequency when performing AGPs, so the H3 hypothesis was formulated, as follows:

*H3*. *There is a negative correlation between wearing disposable coverall when performing aerosol generating procedures (AGPs) on a COVID-19 patient and the occurrence of the following accidents*: *splashing of biological fluids/ respiratory secretions in the eyes; splashing of biological fluids/ respiratory secretions in the oral/ nasal mucosa; splashing of biological fluids/ respiratory secretions on the non idemn skin; puncture/ sting with any material contaminated with biological fluids/ respiratory secretions*.

Although we have found in the literature that disposable gloves can spread infection with the SARS-CoV-2 virus, we started from a positive hypothesis, which underlines the role of disposable gloves in preventing the infection with the virus:

*H4*. *The average number of accidents (SPLASY_EYES*, *SPLASH_ORAL*, *SPLASH_SKIN*, *PUNCTURE_FS) decreases when the use of disposable gloves (AGP_GLOVES) increases when applying the aerosol generating procedures (AGPs) on a COVID-19 patient*.

## 3. Methodology

This study was conducted using a questionnaire translated and adapted from the WHO [13] questionnaire on HCWs exposure risk assessment and management. Our aim was to understand the real impact of specific PPEs used for managing the risk of SARS-CoV-2 virus infection of HCWs exposed to the virus in a healthcare unit by analysing the PPEs used in AGPs for HCWs protection, and against the accidents occurring when performing AGPs. More precisely, data were collected online from the medical staff of the “Sf. Ioan cel Nou” County Emergency Hospital in Suceava in Romania, the confidentiality of responses having been ensured.

As a result, 312 answers were collected between 10.12.2020–19.03.2021. Given the fact that the pandemic generated by the SARS-CoV-2 virus appeared globally in December 2019, and its first effects began to appear in Romania in March 2020, the time period of data collection (until March 2021) enabled us to provide a plausible interpretation of responses due to long time period for analysis of what had happened at the “St. Ioan cel Nou” County Emergency Hospital in Suceava, Romania during the pandemic. This study was given an ethical approval by the “St. Ioan cel Nou” County Emergency Hospital before data collection, an ethical approval from Grigore T. Popa University of Medicine and Pharmacy, Iași, Romania (Approval from 15.06.2020), the collaboration protocol having been signed by three parties–National Authority for Quality Management in Health, Grigore T. Popa University of Medicine and Pharmacy, Iași, and Alexandru Ioan Cuza University of Iași (Approval number 12677/ 27.07.2020). The consent of the respondents in the research was informed, and implicitly. Before completing the questionnaire, was mentioned that the answers are confidential, and that respondents are kindly asked to continue only if they agree to participate in the study.

The instrument includes a complex questionnaire comprising several parts that were applied to medical staff who had been in contact with patients diagnosed with COVID-19. All questions referred to the last 3 months of activity prior to questionnaire completion. Table 1 presents the questions and answers clarifying the methodology used in this research.

**Table 1:** Items and results clarifying research methodology (N = 312).

Question	Code	Items*	Frequency	Valid Percentage	Cumulative Percentage
Gender	GENDER	Male	56.00	17.95	17.95
		Female	256.00	82.05	100.00
**Total**	**-**	**-**	**312.00**	**100.00**	**-**
Category of medical staff:	CATEGORY_STAFF	Doctor /resident	56.00	17.95	17.95
		Nurse	255.00	81.73	99.68
		Other	1.00	0.32	100.00
**Total**	**-**	**-**	**312.00**	**100.00**	**-**
The department where the activity is conducted:	DEPARTMENT	Internal Medicine	7.00	2.24	2.24
		Nephrology	9.00	2.88	5.13
		Gastroenterology	5.00	1.60	6.73
		Cardiology	14.00	4.49	11.22
		Orthopedics–Traumatology	17.00	5.45	16.67
		Urology	5.00	1.60	18.27
		Pediatric surgery and orthopaedics	3.00	0.96	19.23
		Obstetrics–Gynaecology	26.00	8.34	27.56
		Neonatology	10.00	3.21	30.77
		O.R.L.	4.00	1.28	32.05
		Ophthalmology	5.00	1.60	33.65
		Rheumatology	6.00	1.92	35.58
		Psychiatry	8.00	2.56	38.14
		Neurosurgery	8.00	2.56	40.71
		Infectious diseases	23.00	7.37	48.08
		Pneumology	15.00	4.81	52.88
		Chronicles (Palliative care)	14.00	4.49	57.37
		ICU	24.00	7.69	65.06
		ER	85.00	27.25	92.31
		Paediatrics	13.00	4.17	96.47
		Other	11.00	3.53	100.00
**Total**	**-**	**-**	**312.00**	**100.00**	**-**

Source: authors’ contribution.

As it can be seen from the table above, 82.05% and 17.95% of respondents are female and male, respectively. Most respondents are nurses (81.73%) belonging to the following departments: ER (27.25%); Obstetrics—Gynaecology (8.34%); ICU (7.69%) and Infectious diseases (7.37%). The dispersion of answers obtained for 21 departments is quite high for the 312 answers, as we aimed to cover all hospital disciplines that would generate most realistic results.

An open-ended question asking respondents to specify their age was also included into the questionnaire. The average age of medical staff included in the study was 41.75 years.

For all 22 items used in this research and presented in Table 2, the Cronbach’s Alpha coefficient had a value of 0.623, which shows a high reliability of the questionnaire.

**Table 2:** Descriptive statistics for research questions (N = 312).

Question	Code	Items*	Frequency	Valid Percentage	Cumulative Percentage
For each specific PPE item below, indicate how often you have used it when performing aerosol generating procedures on a COVID-19 patient: *					
Disposable gloves	AGP_GLOVES	Always	157.00	98.13	98.13
		Most of the time	2.00	1.25	99.38
		Occasionally	1.00	0.62	100.00
		Rarely	0.00	0.00	100.00
		Never	0.00	0.00	100.00
**Total**	-	-	**160.00**	**100.00**	**-**
Medical masks N95 (or equivalent)	AGP_MASK	Always	143.00	92.86	92.86
		Most of the time	7.00	4.55	97.41
		Occasionally	3.00	1.95	99.36
		Rarely	1.00	0.64	100.00
		Never	0.00	0.00	100.00
**Total**	-	-	**154.00**	**100.00**	**-**
Visor or goggles	AGP_VISOR	Always	145.00	91.19	91.19
		Most of the time	12.00	7.55	98.74
		Occasionally	2.00	1.26	100.00
		Rarely	0.00	0.00	100.00
		Never	0.00	0.00	100.00
**Total**	-	-	**159.00**	**100.00**	**-**
Disposable coverall	AGP_COVERALL	Always	146.00	91.25	91.25
		Most of the time	4.00	2.50	93.75
		Occasionally	8.00	5.00	98.75
		Rarely	2.00	1.25	100.00
		Never	0.00	0.00	100.00
**Total**	-	-	**160.00**	**100.00**	**-**
Footwear protection	AGP_FOOTPROT	Always	152.00	95.00	95.00
		Most of the time	5.00	3.13	98.13
		Occasionally	3.00	1.87	100.00
		Rarely	0.00	0.00	100.00
		Never	0.00	0.00	100.00
**Total**	-	-	**160.00**	**100.00**	**-**
Waterproof apron	AGP_SORT	Always	62.00	40.00	40.00
		Most of the time	20.00	12.90	52.90
		Occasionally	21.00	13.55	66.45
		Rarely	6.00	3.87	70.32
		Never	46.00	29.68	100.00
**Total**	-	-	**155.00**	**100.00**	**-**
*During aerosol generating procedures applied on a patient with COVID-19 (respond only where necessary in the last three months)*:					
You have removed and replaced your PPE in accordance with the protocol (for example, when the surgical mask became wet, did you throw the wet PPE in the trash, did you perform hand hygiene, etc.)?	AGP_RR_PPE	Always	135.00	82.82	82.82
		Most of the time	19.00	11.66	94.48
		Occasionally	3.00	1.84	96.32
		Rarely	1.00	0.61	96.93
		Never	5.00	3.07	100.00
**Total**	-	-	**163.00**	**100.00**	**-**
Did you perform hand hygiene before and after you have touched a patient with COVID-19? Note: Regardless of wearing gloves.	AGP_HANDH_TOUCH	Always	155.00	95.09	95.09
		Most of the time	5.00	3.07	98.16
		Occasionally	3.00	1.84	100.00
		Rarely	0.00	0.00	100.00
		Never	0.00	0.00	100.00
**Total**	-	-	**163.00**	**100.00**	**-**
Did you perform hand hygiene before and after any procedure that requires asepsis conditions (for example: peripheral vascular catheterization, urinary catheterization, intubation etc.)?	AGP_HANDH_ASEPSIS	Always	158.00	96.93	96.93
		Most of the time	4.00	2.45	99.38
		Occasionally	1.00	0.62	100.00
		Rarely	0.00	0.00	100.00
		Never	0.00	0.00	100.00
**Total**	-	-	**163.00**	**100.00**	**-**
Did you perform hand hygiene after touching objects nearby the patient with COVID-19 (bed, door handle etc.)? Note: Regardless of wearing gloves.	AGP_HANDH_OBJECTS	Always	142.00	87.12	87.12
		Most of the time	18.00	11.04	98.16
		Occasionally	3.00	1.84	100.00
		Rarely	0.00	0.00	100.00
		Never	0.00	0.00	100.00
**Total**	-	-	**163.00**	**100.00**	**-**
Were the most touched surfaces decontaminated frequently (at least 3 times a day)?	AGP_SURFACES_DECONT	Always	137.00	84.05	84.05
		Most of the time	20.00	12.27	96.32
		Occasionally	6.00	3.68	100.00
		Rarely	0.00	0.00	100.00
		Never	0.00	0.00	100.00
**Total**	-	-	**163.00**	**100.00**	**-**
*During the period in which you have interacted with a COVID-19 patient for his/ her care*, *in the last 3 months*, *have you had any episodes of accidental exposure to biological fluids/ respiratory secretions*? *If the answer is "Yes"*, *what kind of accident did you have*? *(Answer only if necessary)*					
Splashing biological fluids/ respiratory secretions in the eyes	SPLASY_EYES	-	11.00	-	-
Splashing biological fluids/ respiratory secretions in the oral/ nasal mucosa	SPLASH_ORAL	-	3.00	-	-
Splashing biological fluids/ respiratory secretions on the non idemn skin	SPLASH_SKIN	-	11.00	-	-
Puncture/ sting with any material contaminated with biological fluids/ respiratory secretions	PUNCTURE_FS	-	3.00	-	-
Other	OTHER_ACC	-	0.00	-	-
**Total**	-	-	**28.00**	**-**	**-**
*On a scale of 1 to 5 (1- Not at all*, *5- Very much)*, *please answer the following questions*:					
How satisfied are you with the measures implemented in the health unit where you work?	SATISFY_MEASURES	Very much	157.00	50.32	50.32
		Very	91.00	29.17	79.49
		Moderately	41.00	13.14	92.63
		Slightly	9.00	2.88	95.51
		Not at all	14.00	4.49	100.00
**Total**	-	-	**312.00**	**100.00**	**-**
How would you describe your current access to personal protective equipment?	ACCESS_PPA	Very much	228.00	73.08	73.08
		Very	43.00	13.78	86.86
		Moderately	25.00	8.01	94.87
		Slightly	4.00	1.28	96.15
		Not at all	12.00	3.85	100.00
**Total**	-	-	**312.00**	**100.00**	**-**
How would you describe your current COVID-19 test access?	ACCESS_COVID19_TEST	Very much	173.00	55.45	55.45
		Very	58.00	18.59	74.04
		Moderately	49.00	15.71	89.75
		Slightly	10.00	3.20	92.95
		Not at all	22.00	7.05	100.00
**Total**	-	-	**312.00**	**100.00**	**-**
Are you worried that you may become infected with COVID-19 because of the work you do?	WORRY_INFW	Very much	81.00	25.96	25.96
		Very	50.00	16.03	41.99
		Moderately	49.00	15.71	57.70
		Slightly	45.00	14.42	72.12
		Not at all	87.00	27.88	100.00
**Total**	-	-	**312.00**	**100.00**	**-**
Are you worried that you might spread COVD-19 to your family members?	WORRY_SPREADF	Very much	115.00	36.86	36.86
		Very	49.00	15.71	52.57
		Moderately	43.00	13.78	66.35
		Slightly	36.00	11.53	77.88
		Not at all	69.00	22.12	100.00
**Total**	-	-	**312.00**	**100.00**	**-**
How much has COVID-19 changed your daily routine?	CHANGE_ROUTINE	Very much	157.00	50.32	50.32
		Very	59.00	18.91	69.23
		Moderately	47.00	15.06	84.29
		Slightly	25.00	8.01	92.30
		Not at all	24.00	7.70	100.00
**Total**	-	-	**312.00**	**100.00**	**-**

* Note: Always (as recommended) = wearing PPE more than 95% of the time; Most of the time = from 50% to 95% of the time; Occasionally = wearing PPE from 20% to less than 50% of the time; Rarely = wearing PPE less than 20% of the time. Source: authors’ contribution.

## 4. Results

The study used 312 answers to perform descriptive, correlation and regression analyses. Descriptive analysis was used to provide an overview of PPEs used during AGPs on COVID-19 patients. The actions of medical staff during AGPs applied to patients with COVID-19, the episodes of accidental exposure to biological fluids/ respiratory secretions during interactions with COVID-19 patients, and personnel’ satisfaction with COVID-19 measures implemented in the hospital–were analysed using correlation and regression analyses, which helped us validate the research hypotheses.

The main descriptive results are shown in Table 2. All asked questions covered the last three months at the time of questionnaire application.

Over 50% of respondents reported having taken part in performing AGPs on COVID-19 patients in the previous 3 months. In this regard, the questionnaire included an item filtering the respondents before applying the main questions that helped us obtain valid results for our analysis.

Most respondents reported always having used disposable gloves (98.13%), medical masks N95 (or equivalent) (92.86%), visor or goggles (91.19%), disposable coverall (91.25%) and footwear protection (95.00%) during AGPs. The waterproof apron had been always used by only 40% of staff, while almost 30% had not used it at all during AGPs.

As for the actions that are required when PPE is used, most respondents reported always having done the following: hand hygiene before and after any procedure requiring asepsis conditions (96.93%).

We expected that a higher percentage of respondents would report having always removed and replaced their PPE in line with the protocol (82.82%), and having practised hand hygiene after touching objects in COVID-19 patient proximity (bed, door handle etc., regardless of wearing gloves) (87.12%). We also expected a higher percentage that could indicate that most touched surfaces had been decontaminated, at least, 3 times a day (84.05%). All these expectations appeared since all the preventive actions had been in place in case of COVID-19 infections.

Asked if they had any episodes of accidental exposure to biological fluids or respiratory secretions during the period in which respondents had interacted with COVID-19 patients for their care in the previous 3 months, they mentioned only 28 accidents: 11 accidents with splashing biological fluids/ respiratory secretions in the eyes, 11 accidents with splashing biological fluids/ respiratory secretions on the non-idemn skin, 3 accidents with splashing biological fluids/ respiratory secretions in the oral/ nasal mucosa and 3 accidents with puncture/ sting with any material contaminated with biological fluids/ respiratory secretions. We cannot sort these types of accidents by categories of more or less risky but we are aware of the fact that any type of accident with exposure to COVID-19 infection attracts a risk of COVID-19 infection for anyone not wearing PPE.

During the study (December 2021-March 2022), there were 116 cases of Covid-19 throughout the hospital, of which 60 cases were in the departments involved in the study. Moreover, in the same period, 58 employees from the entire hospital who came into contact with the infected with Covid 19 staff were quarantined, of which 33 employees were coming from the departments participating in the study.

**Fig 1:**
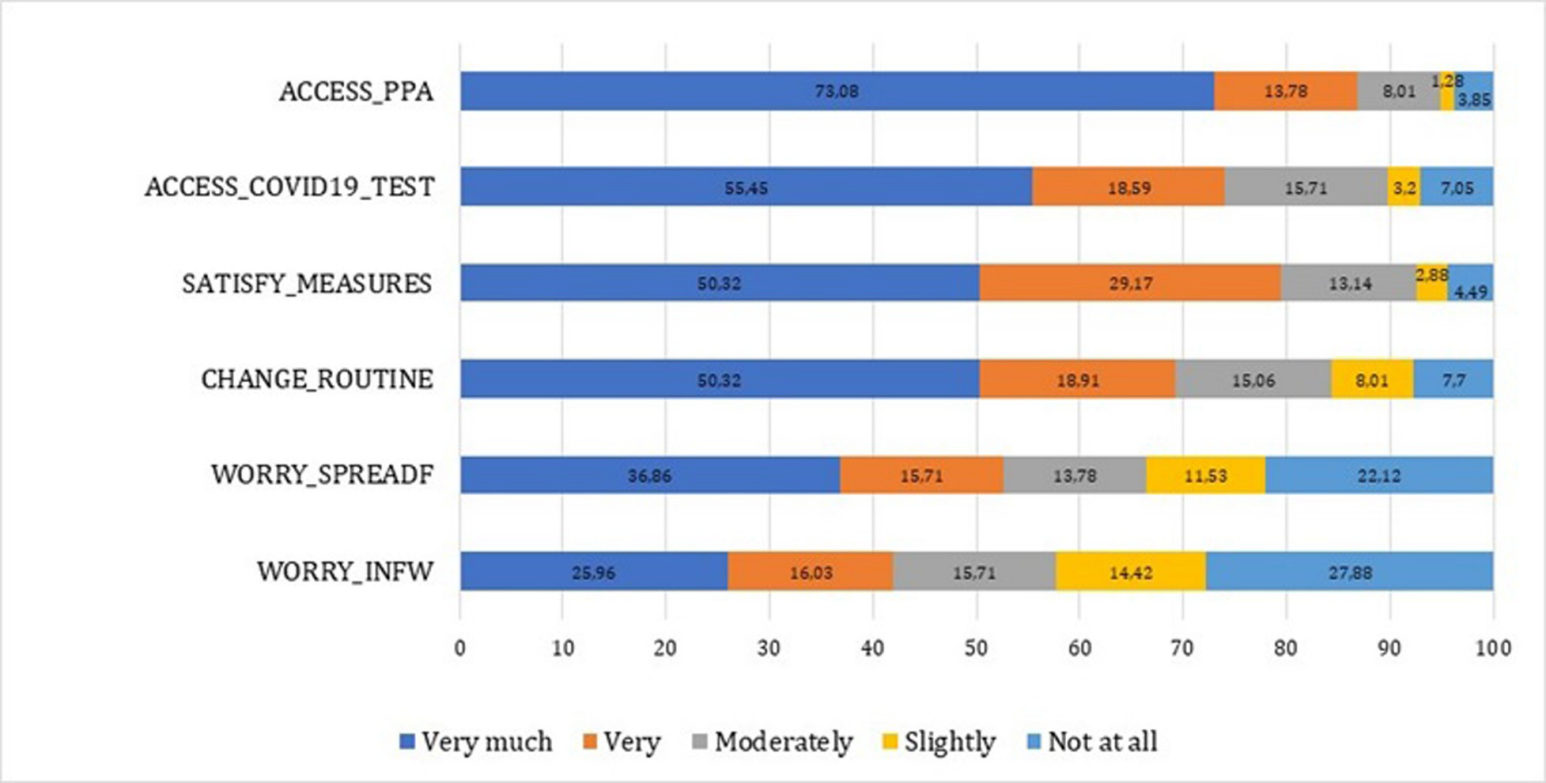
Perceptions of the measures implemented in the hospital and changes in the routine of the HCWs. Source: authors’ contribution.

In the Fig 1 we have also presented the graphical view for the measures implemented in the hospital and the changes in the routine of the HCWs, which complete the descriptive statistics in the previous table.

According to our collected data, personnel’s’ satisfaction with COVID-19 measures implemented in the health unit was ranked as very good by only 50.32% of the respondents, and, at least, as moderate by 92.63%. This means that the measures implemented in the hospital still needed improvements at that time.

The access to PPE was very good for 73.08% of respondents, and, at least, moderate for 94.87%. The COVID-19 test access was very good only for 55.45% of respondents, and, at least, moderate for 89.75%. All in all, these statistics lead us to the idea that improvements are required to have better COVID-19 measures in place in the organization. Also, 57.70% of respondents are, at least, moderately worried that they may become infected with COVID-19 because of the work they do, 66.35% are, at least, moderately worried that they might spread COVD-19 to their family members. Basically, they are more concerned about avoiding the COVD-19 spreading to their family members than about getting the infection themselves. More than half of respondents (50.32%) reported having changed significantly their routine due to COVID-19, and 84.29%, at least moderately.

According to the results presented in Table 3, we have found that there are no differences between male and female participants in the study when using specific PPE items while performing aerosol generating procedures on COVID-19 patients, except the case when the waterproof apron is used (PPE AGP_SORT) (p = 0.015<0.05).

**Table 3:** Kruskal Wallis test for statistically significant differences among PPE items by gender ^a, b^.

	AGP_GLOVES	AGP_MASK	AGP_VISOR	AGP_COVERALL	AGP_FOOTPROT	AGP_SORT
Kruskal-Wallis H	0.514	0.037	1.325	1.486	0.340	5.949
df	1	1	1	1	1	1
Asymp. Sig.	0.474	0.848	0.250	0.223	0.560	0.015

Note: a. Kruskal Wallis Test;

b. Grouping Variable: GENDER.

This result may not necessarily reflect the reality due to low number of male respondents (N = 312 respondents, from which 56 males and 256 females). For this reason, a further research could analyse in detail gender differences in wearing PPE items.

Table 4 shows Hypotheses H1 and H2 validation using the Pearson coefficients.

**Table 4:** Correlation coefficients and associated level of significance. Testing hypotheses H1 and H2.

Variables (PPE) and Pearson Correlation		AGP_GLOVES	AGP_MASK	AGP_VISOR	AGP_COVERALL	AGP_FOOTPROT	AGP_SORT
AGP_GLOVES	Pearson Correlation	1	0.448**	0.633**	0.429**	0.687**	0.025
	p		0.000	0.000	0.000	0.000	0.763
	N		154	159	160	160	154
AGP_MASK	Pearson Correlation		1	0.418**	0.178*	0.332**	0.096
	p			0.000	0.027	0.000	0.239
	N			153	154	154	151
AGP_VISOR	Pearson Correlation			1	0.409**	0.688**	0.065
	p				0.000	0.000	0.425
	N				159	159	153
AGP_COVERALL	Pearson Correlation				1	0.605**	-0.054
	p					0.000	0.510
	N					160	154
AGP_FOOTPROT	Pearson Correlation					1	0.112
	p						0.168
	N						154
AGP_SORT	Pearson Correlation						1
	p						
	N						

Note: ** p < 0.01 (2-tailed);

* p < 0.05 (2-tailed). Source: authors’ contribution.

All Pearson coefficients presented above for the variables mentioned in Hypothesis H1 indicate positive correlations, most of them having medium to strong intensities: there is a positive correlation between wearing disposable coverall (AGP_COVERALL), when performing AGPs on a COVID-19 patient, and wearing disposable gloves (AGP_GLOVES) (Pearson coefficient = 0.429; p = 0.000<0.01), medical masks N95 (or equivalent) (AGP_MASK) (Pearson coefficient = 0.178; p = 0.027<0.05), visor or goggles (AGP_VISOR) (Pearson coefficient = 0.409; p = 0.000<0.01), and footwear protection (AGP_FOOTPROT) (Pearson coefficient = 0.605; p = 0.000<0.01). Therefore, hypothesis H1 has been confirmed:

*H1*. *There is a positive correlation between wearing disposable coverall when performing aerosol generating procedures (AGPs) on a COVID-19 patient and wearing disposable gloves*, *medical masks N95 (or equivalent)*, *visor or goggles*, *and footwear protection*.

The main idea, from which stemmed this hypothesis, was that when medical doctors participate in AGPs and wear a coverall, they also wear disposable gloves, a surgical mask, a visor or goggles and protective footwear, which offer the highest caution equipment for preventing medical staff against COVID-19 infections.

All Pearson coefficients presented in Table 4 for the variables mentioned in hypothesis H2 do not indicate any correlations: there is no correlation between wearing waterproof apron (AGP_SORT), when performing AGPs on a COVID-19 patient, and wearing disposable gloves (AGP_GLOVES) (Pearson coefficient = 0.025; p = 0.763>0.05), medical masks N95 (or equivalent) (AGP_MASK) (Pearson coefficient = 0.096; p = 0.239>0.05), visor or goggles (AGP_VISOR) (Pearson coefficient = 0.065; p = 0.425>0.05), disposable coverall (AGP_COVERALL) (Pearson coefficient = -0.054; p = 0.510>0.05), and footwear protection (AGP_FOOTPROT) (Pearson coefficient = 0.112; p = 0.168>0.05). Therefore, hypothesis H2 has not been confirmed:

*H2*. *There is a positive correlation between wearing waterproof apron*, *when performing aerosol generating procedures (AGPs) on a COVID-19 patient and wearing disposable gloves*, *medical masks N95 (or equivalent)*, *visor or goggles*, *disposable coverall*, *and footwear protection*.

These results are abnormal in our opinion, since the waterproof apron should be completed with disposable gloves, medical masks, visor or goggles, and footwear protection, except disposable coverall. Once a doctor wears a disposable coverall, they cannot wear at the same time a waterproof apron. So, in this case, there should have appeared a significant negative correlation, and it was not noted. We believe the reason that led to the appearance of these statistics that invalidated hypothesis H2 was the high dispersion of the respondents’ declarations on wearing or not of a waterproof apron during AGPs (“Always” frequency = 62.00; “Most of the time” frequency = 20.00; “Occasionally” frequency = 21.00; “Rarely” frequency = 6.00; “Never” frequency = 46.00, as it can be seen in Table 2).

The validation of hypothesis H3 is shown in Table 5 using a correlation analysis.

**Table 5:** Correlation coefficients and associated level of significance. Testing hypothesis H3.

Variables (accidents and PPE) and Pearson Correlation		AGP_COVERALL
SPLASY_EYES	Pearson Correlation	-0.110
	P	0.166
	N	160
SPLASH_ORAL	Pearson Correlation	-0.125
	P	0.115
	N	160
SPLASH_SKIN	Pearson Correlation	-0.178*
	P	0.024
	N	160
PUNCTURE_FS	Pearson Correlation	-0.125
	P	0.115
	N	160

Note: * p < 0.05 (2-tailed). Source: authors’ contribution.

The Pearson coefficients presented in Table 5 for the variables mentioned in hypothesis H3 indicate no correlations between wearing disposable coverall (AGP_COVERALL) when performing AGPs on a COVID-19 patient, and the following accidents: splashing biological fluids/ respiratory secretions in the eyes (SPLASH_EYES) (Pearson coefficient = -0.110; p = 0.166>0.05), splashing biological fluids/ respiratory secretions in the oral/ nasal mucosa (SPLASH_ORAL) (Pearson coefficient = -0.125; p = 0.115>0.05), and puncture/ sting with any material contaminated with biological fluids/ respiratory secretions (PUNCTURE_FS) (Pearson coefficient = -0.125; p = 0.115>0.05).

However, the Pearson coefficients show a very weak negative correlation between wearing disposable coverall (AGP_COVERALL) when performing AGPs on a COVID-19 patient and splashing biological fluids/ respiratory secretions on the non-idemn skin (SPLASH_SKIN) (Pearson coefficient = -0.178; p = 0.024<0.05). It shows that the frequency of accidents, namely, splashing biological fluids/ respiratory secretions on the non-idemn skin (SPLASH_SKIN) lowers when the PPE disposable coveralls are worn often. Consequently, we agree that the disposable coverall is the more efficient type of PPE preventing possible infections with COVID-19.

The H3 hypothesis was formulated starting from the premise that the disposable coverall is the best method for medical staff to protect themselves against any type of accident. However, the correlations between the variables of this hypothesis are mostly statistically insignificant (p>0.05), showing that even if the disposable coverall is worn when AGPs are performed on patients with COVID-19, there is still exposure to the risk of infection. The only protection offered by wearing disposable coverall, in line with our analysis, and as it results from the correlations, is related to splashing biological fluids/ respiratory secretions on the non-idemn skin.

All these being said, the hypothesis H3 has not been confirmed:

*H3*. *There is a negative correlation between wearing disposable coverall when performing aerosol generating procedures (AGPs) on a COVID-19 patient and the following accidents*: *splashing biological fluids/ respiratory secretions in the eyes; splashing biological fluids/ respiratory secretions in the oral/ nasal mucosa; splashing biological fluids/ respiratory secretions on the non idemn skin; puncture/ sting with any material contaminated with biological fluids/ respiratory secretions*.

The validation of hypothesis H4 is shown in Tables 6 and 7 using the regression analysis.

**Table 6:** Model summary. Testing hypothesis H4.

Model	R	R Square	Adjusted R Square	Std. Error of the Estimate
1	0.638^a^	0.408	0.360	0.09560
2	0.638^b^	0.408	0.365	0.09526
3	0.638^c^	0.407	0.369	0.09492
4	0.638^d^	0.407	0.373	0.09461
5	0.637^e^	0.406	0.377	0.09435
6	0.635^f^	0.404	0.379	0.09422
7	0.629^g^	0.395	0.374	0.09453
8	0.620^h^	0.384	0.367	0.09507

a. Predictors: (Constant), AGP_SURFACES_DECONT, AGP_SORT, AGP_RR_PPE, AGP_MASK, AGP_HANDH_ASEPSIS, AGP_COVERALL, AGP_VISOR, AGP_HANDH_OBJECTS, AGP_GLOVES, AGP_HANDH_TOUCH, AGP_FOOTPROT

b. Predictors: (Constant), AGP_SORT, AGP_RR_PPE, AGP_MASK, AGP_HANDH_ASEPSIS, AGP_COVERALL, AGP_VISOR, AGP_HANDH_OBJECTS, AGP_GLOVES, AGP_HANDH_TOUCH, AGP_FOOTPROT

c. Predictors: (Constant), AGP_SORT, AGP_RR_PPE, AGP_MASK, AGP_HANDH_ASEPSIS, AGP_COVERALL, AGP_VISOR, AGP_GLOVES, AGP_HANDH_TOUCH, AGP_FOOTPROT

d. Predictors: (Constant), AGP_SORT, AGP_RR_PPE, AGP_MASK, AGP_HANDH_ASEPSIS, AGP_COVERALL, AGP_GLOVES, AGP_HANDH_TOUCH, AGP_FOOTPROT

e. Predictors: (Constant), AGP_SORT, AGP_MASK, AGP_HANDH_ASEPSIS, AGP_COVERALL, AGP_GLOVES, AGP_HANDH_TOUCH, AGP_FOOTPROT

f. Predictors: (Constant), AGP_SORT, AGP_MASK, AGP_HANDH_ASEPSIS, AGP_GLOVES, AGP_HANDH_TOUCH, AGP_FOOTPROT

g. Predictors: (Constant), AGP_MASK, AGP_HANDH_ASEPSIS, AGP_GLOVES, AGP_HANDH_TOUCH, AGP_FOOTPROT

h. Predictors: (Constant), AGP_HANDH_ASEPSIS, AGP_GLOVES, AGP_HANDH_TOUCH, AGP_FOOTPROT

Source: authors’ contribution.

**Table 7:** Coefficients for the final model (Model no. 8). Testing hypothesis H4.

No.	Model	B	Std. Error	Beta	t	p value
8	(Constant)	0.869	0.215		4.04	0
	AGP_GLOVES	-0.232	0.057	-0.385	-4.076	0
	AGP_FOOTPROT	-0.069	0.036	-0.189	-1.919	0.057
	AGP_HANDH_TOUCH	-0.155	0.035	-0.427	-4.395	0
	AGP_HANDH_ASEPSIS	0.286	0.049	0.546	5.844	0

Note: Dependent Variable = Mean_accidents. Source: authors’ contribution.

The dependent variable in the model is the average number of accidents reported by respondents, in numbers, during AGPs on patients with COVID-19. Initially, 11 independent variables were introduced into the model. These are listed in detail in Table 2 and include PPE and preventive protective measures against COVID-19: AGP_SURFACES_DECONT, AGP_SORT, AGP_RR_PPE, AGP_MASK, AGP_HANDH_ASEPSIS, AGP_COVERALL, AGP_VISOR, AGP_HANDH_OBJECTS, AGP_GLOVES, AGP_HANDH_TOUCH, AGP_FOOTPROT. From these 11 predictors, using Backward method, only 4 predictors remained in the final model (Model no. 8): AGP_HANDH_ASEPSIS, AGP_GLOVES, AGP_HANDH_TOUCH, AGP_FOOTPROT.

The eighth variant of the model is the best, with R of 0.620, R^2^ of 0.384, adjusted R^2^ of 0.367, and an estimated Std. Error of 0.09507. As it can be seen, the value of R (0.620) indicates a relatively good correlation between the average number of accidents, as opposed to the elements of PPE, and preventive protective measures against COVID-19. The values of R^2^ (0.384) and adjusted R^2^ (0.367) indicate a relatively low to average proportion of the variation in the dependent variable explained by the regression model.

The final model (Model no. 8) is explained in proportion of 36.7%, which is a realistic percentage, if we consider the number of reported accidents (only 28 in total, as it is shown in Table 2).

The average number of accidents decreases as the coefficients of AGP_GLOVES, AGP_FOOTPROT and AGP_HANDH_TOUCH grow. In other words, as disposable gloves and footwear protection are used in performing AGPs on patients with COVID-19, and hand hygiene is done before and after touching a patient with COVID-19 (regardless of wearing gloves), the number of accidents should decrease.

All these being said, the hypothesis H4 has been confirmed:

*H4*. *The average number of accidents (SPLASY_EYES*, *SPLASH_ORAL*, *SPLASH_SKIN*, *PUNCTURE_FS) decreases when the use of disposable gloves (AGP_GLOVES) increases during performing aerosol generating procedures (AGPs) on a COVID-19 patient*.

The problem in this model is that the results indicate that the number of accidents increases as the coefficient of AGP_HANDH_ASEPSIS increases. So, as long as there is hand hygiene before and after any procedure requiring asepsis conditions (for example: peripheral vascular catheterization, urinary catheterization, intubation, etc.), the number of accidents increases. We think this could be possible only if hygiene before and after any procedure requiring asepsis conditions was not properly done. Another reason for such an output could also be the low number of accidents (28, detalis in Table 2), which could statistically explain this abnormal last result.

## 5. Conclusions

This study started from the results reported by earlier studies broken down into three research lines: AGPs to COVID-19 patients, PPE during AGPs on COVID-19 patients, and accidents during AGPs involving COVID-19 patients and ways of protecting HCWs. Further, we presented PPEs used during AGPs for the protection of HCWs and against accidents that appear when performing AGPs, after analysing 312 confidential answers collected from the medical staff of the “Sf. Ioan cel Nou” County Emergency Hospital, Suceava, Romania. For this study, the ethical approval was given.

The main results indicate good compliance with the recommendations for managing and lowering the risk of exposure. Most respondents always used, during AGPs, disposable gloves (98.13%), medical masks N95 (or equivalent) (92.86%), visor or googles (91.19%), disposable coverall (91.25%) and footwear protection (95.00%). The waterproof apron was always used by only 40% of staff, and almost 30% not using it at all during AGPs. More than half of respondents (50.32%) reported having changed very much their routine because of COVID-19, and 84.29%, at least, moderately.

We derived from the WHO recommendations [13] no less than 11 independent factors (details in Table 6) related to risk exposure management, defined as hygienic actions or wearing of protective equipment, and used them as variables in a regression analysis. We proposed 4 hypotheses, from which, 2 have been validated (H1 and H4), and 2 have not (H2 and H3). We have found a positive correlation between wearing disposable coverall when performing AGPs on COVID-19 patients, and wearing disposable gloves, medical masks N95 (or equivalent), visor or goggles, and footwear protection, and the fact that the average number of accidents (SPLASY_EYES, SPLASH_ORAL, SPLASH_SKIN, PUNCTURE_FS) decreases when the use of disposable gloves grows during performing AGPs on COVID-19 patients.

We found no positive correlation between wearing a waterproof apron when performing AGPs on COVID-19 patients, and wearing disposable gloves, medical masks N95 (or equivalent), visor or goggles, disposable coverall, and footwear protection. We believe this result has a statistical reason, namely the high dispersion of the respondents’ declarations on wearing or not of a waterproof apron during AGPs. Thus, we consider hypothesis H2 a future interesting investigation topic. Also, we found no negative correlation between wearing disposable coverall when performing AGPs on COVID-19 patients, and the analysed accidents.

The only protection offered by a disposable coverall, as it results from our analysis, is related to splashing biological fluids/ respiratory secretions on a non-idemn skin. Also, the results show that as disposable gloves and footwear protection are used in performing AGPs on patients with COVID-19 and hand hygiene is also done before and after touching a patient with COVID-19 (regardless of wearing gloves), the number of accidents should decrease.

According to the results, there were no differences between male and female participants when using specific PPE items while performing AGPs on COVID-19 patients, except the case when the waterproof apron is used. For this reason, a further research could analyse in detail gender differences in wearing PPE items.

## 6. Discussions

We found in the literature that COVID-19 could be spread through aerosol/ aerated solids, fluid from human secretions, droplets from normal breathing, coughing, sneezing and surface contact, and, thus, it is considered to be very contagious [3], and that nurses are extremely exposed to COVID-19 since they have a direct contact with COVID-19 patients, especially in the context of AGPs [9, 12, 31]. In managing the health workers exposure risk, and to avoid any contamination with COVID-19 during aerosols, the use of PPE is recommended for eye, face and head protection, together with reusable equipment (glasses, visors or face shields) [17, 20]. Goggles are extremely important in protecting the exposed conjunctiva from infected droplets and aerosols from patients, while aprons, gowns and coveralls offer full protection to HCWs [10, 25]. Glove usage can bring a false safety feeling, and Al Youha el al. (2021) found a positive correlation between wearing gloves by nurses, and COVID-19 infections to HCWs [6].

In line with the exposure risk management recommendations, in our study we expected that a higher percentage of respondents had taken off and replaced their PPE, in accordance with the protocol (82.82%), and had always performed hand hygiene after touching an object found in close proximity to a COVID-19 patient (bed, door handle etc., regardless whether gloves are worn or not) (87.12%).

The present research was carried out at the “Sf. Ioan cel Nou” Hospital in Suceava, Romania, which was the first outbreak of infection in Romania. Thus, the novelty of our research consisted in suggestions for risk management, extremely complicated at the intersection of three large areas of analysis: PPE, AGP, Covid-19.

According to Girma et al., 2020, HCWs’ risk perceptions may be a factor that could influence the wearing of PPE [36]. This study did not include this factor in its analysis. Also, we did not consider the hand hygiene goals set in the analysed unit, so useful in this pandemic [37, 38]. Another limitation of the study is related to the time span covered when asking the questions: three months into the past. This time interval was set by us considering the possibility of respondents to recall events, decreasing as time span increases, and the need to get relevant statistical figures about expected rare contamination events, increasing as the time span increases. Hence, we consider that studies with a different time span can lead to different results.

## 7. Future prospect and application of this study

Previous studies adressed the challenge to further analyzes related to PPEs [37], others pointed out that PPE is generally used at an acceptable level [36], while others focused on the importance of PPE wearing by HCWs [20, 22, 30].

The results presented in our study are useful for risk management, when the following are put together: PPE, AGP, and Covid-19. Given the fact that AGPs are procedures through which infections can easily spread, and the management of any hospital wants to prevent the spread of Covid-19 infections, we conclude that only with adequate PPEs, HCWs could avoid accidents.

Because we found no positive correlation between wearing a waterproof apron when performing AGPs on COVID-19 patients, and wearing disposable gloves, medical masks N95 (or equivalent), visor or goggles, disposable coverall, and footwear protection, we consider this result a starting point for future investigation. Also, given the fact that between wearing disposable coverall when performing AGPs on COVID-19 patients, and the analysed accidents no correlation were found, we expect further research results related to this conclusion.

Future research perspectives on the mix composed by PPE, AGP, and Covid-19 could also be analyzed in the post pandemic times. An interesting comparative analysis between the using of PPE during AGPs in Covid and non-Covid times would be also useful for hospitals management in the future.

## Supporting information

S1 File (SAV)
