# Correction: Exposure risk management: Personal protective equipment and the risk of accidents occurring during aerosol generating procedures applied to COVID-19 patients

**DOI:** 10.1371/journal.pone.0315104

**Published:** 2024-12-03

**Authors:** Ștefan Andrei Neştian, Silviu-Mihail Tiţă, Elena-Sabina Turnea, Oana Stanciu, Vladimir Poroch

An additional affiliation is missing for the fifth author. Vladimir Poroch is also affiliated with the PhD Candidate, Doctoral School of Economics and Business Administration, Alexandru Ioan Cuza University, Iași, Romania.
